# Incidence of avian malaria in hummingbirds in humid premontane forests of Pichincha Province, Ecuador: A pilot study

**DOI:** 10.14202/vetworld.2021.889-896

**Published:** 2021-04-13

**Authors:** Claudia S. Abad, Markus P. Tellkamp, Isidro R. Amaro, Lilian M. Spencer

**Affiliations:** 1 Department of Biology, School of Biological Sciences and Engineering, Yachay Tech University, Urcuquí, Ecuador; 2 Department of Mathematics, School of Mathematical and Computational Sciences, Yachay Tech University, Urcuquí, Ecuador; 3 Department of Cell Biology, Simón Bolívar University, AP 89000 Caracas, Venezuela

**Keywords:** avian malaria, humid forest, hummingbirds, Pichincha, *Plasmodium*

## Abstract

**Background and Aim:**

Avian malaria is a tropical disease caused by protozoans of the genera *Plasmodium* and *Haemoproteus*. As a nonlethal disease, avian malaria can affect the lifespan and reproductive rate of birds. If there is a differential effect depending on bird species, then this disease might have a significant effect on avian biodiversity. The current study aimed to determine the incidence of *Plasmodium* in hummingbirds in humid premontane forest areas.

**Materials and Methods:**

Blood samples (n=60) were collected from hummingbirds from two areas (Santuario de Aves Milpe and Hacienda Puyucunapi) of Pichincha Province, Ecuador. Prevalence and parasitemia were determined by microscopic examination of blood smears stained with Giemsa reagent. Both study sites are part of a 1000 m elevational gradient; hence, elevation was used as a predictor variable for prevalence and parasitemia levels in a Mann–Whitney U-test. This test was also used to test for a sex bias.

**Results:**

This study reports on a total of 12 bird species that inhabit both study sites. At Milpe, the lower elevation site, a prevalence of 100% was recorded, whereas at Puyucunapi, the prevalence was 96%. The combined prevalence was 97%. Elevation and sex did not influence prevalence nor parasitemia in hummingbirds.

**Conclusion:**

This study does not suggest a significant elevation or sex bias on prevalence and parasitemia in hummingbirds.

## Introduction

Parasitism is a species interaction that is pervasive worldwide, with over 31,000 protozoan parasites already described and more than three-quarters yet to be reported. The research that has been conducted to date on protozoan species is usually tied to their medical and economic significance. Hence, species parasitizing mammals have been more widely studied than those parasitizing birds and reptiles [1]. Malaria is a pervasive parasitic disease caused by protozoans of the phylum Apicomplexa, as defined by Levine in 1970 [2]. Avian malaria was discovered in 1885, and from that point on, it has played a significant role in understanding human malaria. Avian malaria has served as a model to explain how malaria parasites are transmitted and been used to develop new medications to treat the disease. Recognizing and describing avian malaria lineages have led to the acknowledgment of their prevalence, diversity, and distribution worldwide. For that reason, studies on avian malaria have thrived in the past 60 years, and avian malaria remains a trending topic for research [3]. *Plasmodium* and *Haemoproteus* are genera of hemosporidians, blood-borne protozoan parasites, and the etiological agents of malaria in birds [1,2]. At present, there are more than 250 species of hemosporidians described thanks to parasitological diagnosis [4]. *Plasmodium* is a parasite transmitted by mosquitoes while *Haemoproteus* is transmitted by biting midges and louse flies [5]. *Plasmodium* and *Haemoproteus* are paraphyletic, have a common ancestor, and share many biological features; therefore, they can be easily confused [6]. For example, the gametocytes of both genera are almost indistinguishable [1]. Furthermore, malaria pathogens infect not only the blood but also several other organs in birds [7].

**Figure-1:**
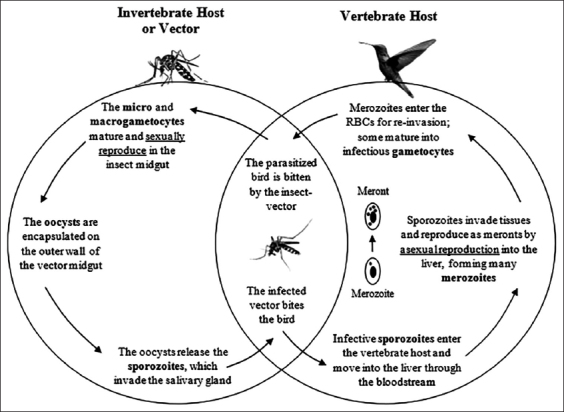
Hemosporidian general life cycle, exemplified by *Plasmodium*. When the insect vector ingests infected blood with microgametocytes and macrogametocytes, the sexually reproductive stage occurs in the midgut of the insect, producing mobile zygotes that penetrate the epithelial cells of the midgut and originate the oocysts. This oocyst releases the sporozoite stage, which migrates and accumulates in the salivary glands of the invertebrate host making it possible to infect another vertebrate host with its next bite. The sporozoites can infect several organs such as the heart, spleen, lung, liver, kidneys, and brain. Then, an asexual reproduction or merogony occurs; the merozoite multiplies within red blood cells (RBCs) to form the meront. The final maturation process of the meront is achieved by releasing hundreds of merozoites, which can parasitize diverse organs or other RBCs.

Avian malaria has the potential to exert strong selective pressure on birds since it is frequently virulent [3]. Some of the effects on bird physiology during the initial acute phase of the infection are anemia, lethargy, and appetite loss [8]. As they are widely distributed around the world, avian malaria parasites have similar patterns of diversity as their hosts. In general, *Haemoproteus* is more diverse than *Plasmodium* except in South America, where *Plasmodium* is more prevalent and widespread [9]. The protozoans causing malaria are obligate heteroxenous protists; therefore, they need more than 1 host to complete their life cycle. Thus, the life cycle of malaria hemoparasites involves two hosts, the invertebrate host or insect vector and the vertebrate host, a bird in the case of avian malaria. The general life cycle for both *Plasmodium* and *Haemoproteus* is explained in Figure-1. However, there are some differences in the infection process of both genera. For example, they use different dipteran families as vectors; *Plasmodium* utilizes blood-sucking mosquitoes (Culicidae), while *Haemoproteus* utilizes biting midges (Ceratopogonidae) and louse flies (Hippoboscidae) [7,10]. Another significant difference is that *Haemoproteus* meronts are only found in the internal organs of the vertebrate host, while *Plasmodium* parasites can be found in red blood cells as merozoites, meronts, and gametocytes [1]. Finally, these genera have a similar life cycle to *Leucocytozoon*, the third genus of avian hemosporidian parasites; however, this genus is transmitted by blackflies (Simuliidae) and does not cause malaria but causes pathologies in birds [10-12]. Moreover, *Leucocytozoon* are not generally found in wild birds, as they are known to primarily affect poultry such as ducks and turkeys [13].

Hummingbirds are birds of the family Trochilidae, which is endemic to the American continents and can be parasitized by both *Plasmodium* and *Haemoproteus*. However, to date, hummingbirds have not been well studied with regard to avian malaria [4,14]. Therefore, we investigated avian malaria in premontane forests, which harbor a great abundance and diversity of hummingbirds [15]. Trochilidae consist of over 300 species and are a good option for comparative studies on the impact of avian malaria on avian diversity due to the great number of species inhabiting diverse habitats. In addition, several physiological features, such as high metabolic rates, ability to enter torpor, tolerance to hyperglycemia, and an efficient circulatory system [16-18], allow hummingbirds to have a very different, active lifestyle compared to that of other birds. Finally, these small birds are of concern because they provide several essential environmental services, such as pollination. Therefore, understanding how malaria affects hummingbirds and what species might be endangered by the disease is essential for their protection and conservation [4].

The aim of this study was to determine the degree of infection of hummingbirds by avian malaria in humid premontane. We chose two sites with a 1000 m elevational difference to test whether prevalence and parasitemia were different along the elevational gradient since the infection affects the reproductive rate of bird species [19]. Given that only females incubate eggs, we also tested for a sex bias in these locations.

## Materials and Methods

### Ethical approval

This study was approved by Mindo Cloudforest Foundation as it follows international guidelines, such as the Guidelines of the Use of Wild Birds in Research by the Ornithological Council. Blood samples were collected as per standard sample collection methods without any harm or stress to the hummingbirds.

### Sampling period and areas

The samples were collected during October 2018-February 2020. Blood sampling was performed at two different reserves managed by the Mindo Cloudforest Foundation in Pichincha Province: The Milpe Bird Sanctuary located in San Miguel de Los Bancos (0°02’12.9’’N 78°52’12.8’’ W, 1150 m.a.s.l.) and Hacienda Puyucunapi located near Nanegalito (0°01’33.5’’N 78°41’48.5’’ W, 2000 m.a.s.l.). The study sites have a 1000 m elevational gradient. They have a similarly humid climate with an annual average temperature of approximately 16°C, an annual average relative humidity of 80%, and an average annual cumulative rainfall of approximately 2525 mm across both areas [20-24]. Moreover, there are permanent streams and stagnating water sources at both locations [25]. A total of 60 birds were captured, 13 in the Milpe area and 47 at Puyucunapi.

### Bird capture and blood sampling

For this study, all hummingbirds (family Trochilidae) were captured using mist nets. Birds were marked or their distinguishing characteristics were recorded such that recaptures were not resampled. First, each bird was weighed (g) and standard measurements, including exposed culmen, tarsometatarsus, tail, and wing length [26], were taken (mm). Other data collected included the presence and state of the brood patch, condition of the cloaca, fat accumulation, muscle volume, and molting presence [27]. Due to hummingbirds’ small body size, blood smears were performed by taking a drop of blood from the metatarsal vein [28]. The drop was placed on a slide, and a blood smear was performed immediately using a spreader slide (coverslip). A duplicate was made for each sample. Once the birds were identified and blood samples were taken, the birds were checked to ensure that bleeding had stopped and were then released. The blood smears were air-dried, fixed with absolute methanol for 3 min, and wrapped in paper towels for transport.

### Specimen staining and parasitological diagnosis

**Figure-2:**
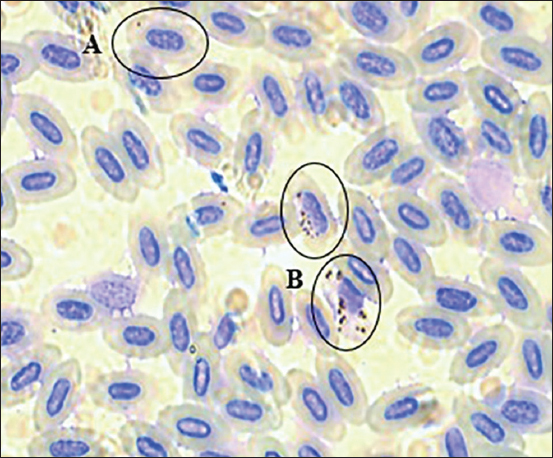
Light microscopy image of *Plasmodium*-infected erythrocytes from an adult Collared Inca (*Coeligena torquata*). The circles demarcate the area where the merozoites and meronts are observed. (A) Erythrocyte parasitized with a *Plasmodium* merozoite. (B) Erythrocytes parasitized with *Plasmodium* meronts. The photography was taken using the professional light optical microscope Leica model DM3000 and 100×.

Samples were stained with Giemsa reagent diluted 1:5 in phosphate-buffered saline at the laboratory at Yachay Tech University [28,29]. Each smear was covered with Giemsa solution for 10 min. Then, they were delicately washed with tap water and placed vertically to air dry [29-31]. Once dry, the stained samples were observed under an optical Leica DM300 microscope for parasitological diagnosis. The samples were observed with an amplification of 100× using immersion oil. An optical field was chosen and the parasitemia percentage was obtained by counting the total number of blood cells and the parasitized red blood cells. The parasitemia percentage was calculated as the number of parasitized red blood cells per 100 cells [1,7,32]. An average of 200 erythrocytes was counted per field of view, and 10 fields were examined per slide. The blood smears were photographed using a professional light optical Leica model DM3000 microscope (Figure-2). In addition, the total infection rate was obtained by dividing the total number of infected individuals by the total number of sampled specimens [33].

### Statistical analysis

A Wilk–Shapiro test was performed to test for normality of the parasitemia data. Since normality was not supported, a non-parametric Mann–Whitney U-test was used to compare the independent samples from Milpe and Puyucunapi. This test is the non-parametric equivalent of the Student’s t-test. Both tests can be considered analogous since their main objective is to confirm or deny the existence of statistically significant differences between the two groups to be studied [34]. In addition, a Mann–Whitney U-test was performed to test for sex bias in parasitemia. For this analysis, 37 individuals were considered because the sexes of the other 23 could not be determined. Among the sexed individuals, 19 were female and 18 were male. R-Studio (2013) version 3.0.1 was used for statistical analyses [35].

## Results

A total of 60 birds from 12 species of hummingbirds were captured at the two study locations. Of these, 47 were captured at Puyucunapi and 13 at Milpe. Therefore, the sample sizes used for statistical analysis were 60 (n=60), 47 for Puyucunapi (Table-1) and 13 for Milpe (Table-2). Of the two studied areas, Puyucunapi had the greatest a-diversity with a total of 10 different bird species (Table-1), while in the Milpe area, two different bird species were captured (Table-2).

**Table-1:** Mean parasitemia percentages per species at Puyucunapi.

Species number	Species	Common name	Number of individuals	Sex	Mean parasitemia (%)
1	*Adelomyia melanogenys*	Speckled hummingbird	1	Unk	5
2	*Aglaiocercus coelestis*	Violet-tailed Sylph	7	2 F, 5 M	6
3	*Boissonneaua flavescens*	Buff-tailed coronet	4	4 Unk	5
4	*Coeligena torquata*	Collared Inca	1	M	4
5	*Coeligena wilsoni*	Brown Inca	13	1 F, 12 Unk	6
6	*Heliodoxa imperatrix*	Empress brilliant	2	2 F	9
7	*Heliodoxa rubinoides*	Fawn-breasted brilliant	13	9 F, 4 M	4
8	*Ocreatus underwoodii*	White-booted racket-tail	1	F	1
9	*Phaethornis syrmatophorus*	Tawny-bellied hermit	3	3 Unk	2
10	*Urosticte benjamini*	Purple-bibbed whitetip	2	1 F, 1 M	5

F=Female, M=Male, and Unk=Unknown

**Table-2:** Mean parasitemia percentages per species at Milpe.

Species number	Species	Common name	Number of individuals	Sex	Mean parasitemia (%)
1	*Heliodoxa jacula*	Green-crowned brilliant	7	1 F, 5 M, 1 Unk	5
2	*Thalurania fannyi*	Green-crowned wood nymph	6	2 F, 2 M, 2 Unk	3

F=Female, M=Male, and Unk=Unknown

**Figure-3:**
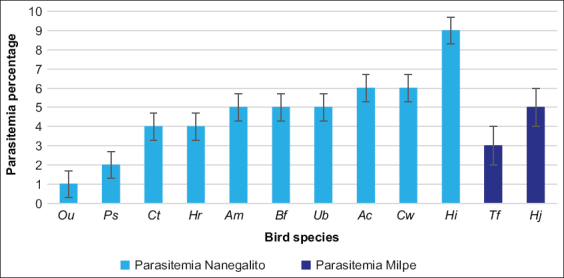
Parasitemia percentage in the different bird species captured at the two sampling areas, Puyucunapi (light blue bars) and Milpe (dark blue bars). The abbreviations for the birds’ species names are *Ocreatus underwoodii* (*Ou*), *Phaethornis syrmatophorus* (*Ps*), *Coeligena torquata* (*Ct*), *Heliodoxa rubinoides* (*Hr*), *Adelomyia melanogenys* (*Am*), *Boissonneaua flavescens* (*Bf*), *Urosticte benjamini* (*Ub*), *Aglaiocercus coelestis* (*Ac*), *Coeligena wilsoni* (*Cw*), *Heliodoxa imperatrix* (*Hi*), *Thalurania fannyi* (*Tf*), and *Heliodoxa jacula* (*Hj*).

Both localities had different species; no species occurred in both sampling areas (Figure-3). At Puyucunapi, 45 of the 47 individuals were infected, representing 96% of the sample. The two individuals that did not present any signs of malaria infection were two females of *Heliodoxa rubinoides* species. In addition, the highest level of parasitemia was 9% which was found in *Heliodoxa imperatrix* (Figure-3). On the other hand, at Milpe, all 13 birds were infected, that is, a prevalence of 100%. The highest parasitemia level at this location was 5% corresponding to *Heliodoxa jacula* (Figure-3). Finally, the difference between the mean parasitemia percentage of both localities was very small since Milpe had an average parasitemia of 4% and Puyucunapi had an average parasitemia of 5%.

The Wilk–Shapiro test suggested that the data were not normally distributed (p=0.01504) [36]. Given that the data did not follow a normal distribution, a Mann–Whitney U-test was performed to compare parasitemia at the two sites. The results were non-significant (p=0.3181) [34]. On the other hand, another Mann–Whitney U-test suggested that there was no sex bias with regard to parasitemia levels (p=0.1122).

## Discussion

There are few studies concerning the avian hemosporidians responsible for malaria in Latin America, Ecuador, included, with some reports in the Galapagos Islands [37-39]. Therefore, the humid premontane forest of Pichincha Province is a location of interest. Hummingbirds (family Trochilidae), to date, have not been well studied in the context of avian malaria [4,14], and our work is the first report on malaria in hummingbirds from Northwest Ecuador. The scant literature suggests that *Haemoproteus* infections are higher in hummingbirds than in passerine birds [4,10,40,41], with the first studies reporting pathologies similar to malaria in hummingbirds 70 years ago, and those confirming avian malaria in Trochilidae as recent as 15 years ago [14,42].

Some of the existing studies concerning *Haemoproteus* infections in hummingbirds include Bradshaw and collaborators, who studied the presence and prevalence of *Haemoproteus archilochus* in two species of California hummingbirds, *Archilochus alexandri* and *Calypte anna*. Blood samples were mainly collected from three locations of Central California. A total of 261 specimens were sampled in 1.5 years. Their results indicate a relatively low *H. archilochus* prevalence with 4.76% in *C. anna* and 37.84% in *A. alexandri* [4]. Another study concerning free-ranging California hummingbirds was reported by Magagna *et al*. [43] in 2019. However, they focused on several pathologies affecting the Trochilidae family and therefore only slightly focused on malaria caused by *Haemoproteus* spp.

The first report of avian hemosporidians in hummingbirds in Ecuador, from both the *Haemoproteus* and *Plasmodium* genera, was reported in 2014 by Harrigan *et al*. [42]; samples were collected for 5 years, from 1999 to 2004, from several locations of the North Andes in Ecuador. Harrigan *et al*. [42] found a great diversity of parasite lineages in the Trochilidae family, with 12 lineages in 11 hummingbird species. In addition, the authors suggested that elevation and mean annual temperature was among the main factors influencing avian malaria prevalence within the ecological gradients they studied in the North Andes of Ecuador. They reported higher prevalences of *Haemoproteus*, *Plasmodium*, and *Leucocytozoon* at higher elevations and lower temperatures, hence supporting the results of the statistical analysis concerning elevation performed in the current study [42]. Another study on hummingbirds in Ecuador was performed by Moens *et al*. [44] in Podocarpus National Park located in Southeast Ecuador. The authors sampled 169 hummingbirds from 19 species and 736 birds of 112 passerine and non-passerine species and evaluated them using light microscopy and polymerase chain reaction. They found four *Haemoproteus* lineages from 50 hummingbirds from 11 species. Twenty-four individuals were also infected with *Leucocytozoon* spp., but none of the individuals were coinfected with *Plasmodium*. Moens *et al*. [44] showed through this study that *Haemoproteus* diversity and specialization were low in hummingbird species of the Ecuadorian Andes. Finally, the authors suggested that although *Haemoproteus* parasites are generalists, their prevalence was higher in hummingbirds than in birds that do not belong to the Trochilidae family. In addition, some studies show that hummingbirds are more susceptible to several pathologies, including avian malaria, because of their high metabolic requirements [44,45]. Hence, future research on the effects of avian malaria in the Trochilidae family is fundamental since they provide several essential environmental services, such as pollination and control of insect species [4].

This study reports on a total of 12 hummingbird species, two of which were from the Milpe area, which represents the lower elevation of the two examined sites. The most parasitized species in this region was *H. imperatrix* with a parasitemia rate of 9% while the least parasitized species was *Ocreatus underwoodii*, with a parasitemia rate of 1%. The prevalence of *Plasmodium* in the two studied areas, Puyucunapi and Milpe, was high, at 96% and 100%, respectively, as expected for areas with a humid forest and the presence of bodies of water [20-24]. These results are consistent with the literature since there is evidence that supports that “avian hemosporidians extend upslope to the limit of available bird habitat [33].” In other words, a higher elevation does not imply a lower prevalence, and there is evidence that the opposite is occurring [33,42].

Pulgarín *et al*. [40] sampled 244 birds in 15 different locations in Colombia. Of the total number of individuals sampled, only 34 presented infections by hemosporidian parasites. Their results suggest that climatic factors such as the mean annual cloud frequency and mean annual precipitation can influence parasite prevalence, but only slightly, which is consistent with the small prevalence variation between the Milpe and Puyucunapi sites in our study. Doussang *et al*. [46] collected samples in 75 different locations of Central and South America. A total of 1317 samples were collected in countries such as Costa Rica, Colombia, Peru, Bolivia, Uruguay, Chile, and Argentina. The total prevalence of both *Plasmodium* and *Haemoproteus* in Central and South America was 25%, with the lowest prevalence reported in Colombia. The authors also found that *Plasmodium* prevalence increased at lower altitudes while *Haemoproteus* prevalence increased at higher elevations. This finding could support the slightly higher prevalence of *Plasmodium* spp. (100%) at the lower studied site, Milpe, compared to that of Puyucunapi (96%) in the current study. Finally, Cuevas *et al*. [41] performed a study in 18 temperate forest localities in Chile. The total prevalence was 28.3% with 146 of 516 birds infected by hemosporidians. An important outcome of this work was the suggestion that prevalence is positively related to host abundance which is useful for future research.

In the current study, meronts were observed when examining blood smears to identify *Plasmodium* spp. parasites. However, they were not found in all samples. In general, the parasite stages found during the microscopic examination in this work were merozoites and meronts. Microgametocytes and macrogametocytes were not observed. As mentioned before (see Introduction), *Haemoproteus* meronts are only found in the internal organs of the vertebrate host, while *Plasmodium* parasites can be found in red blood cells in both sexual and asexual forms. Therefore, only gametocytes are usually observed in erythrocytes infected by *Haemoproteus* lineages [1,10]. For that reason, *Haemoproteus* parasites were not observed under the microscope in the parasitological diagnosis in this study despite it is usually more common than *Plasmodium* in hummingbirds [4,10,40,41]. Moreover, morphologies similar to those reported by other authors for *Haemoproteus* gametocytes were not observed [7,10].

Avian malaria goes from asymptomatic to lethal in domestic, wild, and captive birds [7,47]. Therefore, it has the potential to cause harmful effects on bird fitness because it is frequently virulent to hosts [3,48]. Some of the effects on bird physiology during the initial acute phase of the infection are anemia, lethargy, and appetite loss [8]. Moreover, *Plasmodium* can also provoke pronounced decreases in red blood cell number, brain bleeding, and edema. The spleen and liver are also affected by this pathogen, leading to enlargement and even necrosis on some occasions. Nonetheless, birds that live in areas where malaria is endemic are usually not mortally affected, as is the case in both studied humid forest locations of Pichincha Province [3,49]. In addition, the negative effects of avian malaria on bird physiology might influence bird biodiversity through differential effects on life span and reproductive success [47,50]. Therefore, avian malaria represents a potential threat to avian communities in species rich Neotropics [51,52]. *H. imperatrix*, an endemic species on the western slopes of the West Andes in Ecuador and Colombia, had a particularly high level of parasitemia in this study. Does this high level increase its risk of extirpation? Conversely, is a species with low parasitemia, such as *O. underwoodii*, any safer?

Finally, avian malaria was detected and diagnosed by microscopic examination of Giemsa-stained blood smears due to the advantages associated with this technique, such as its simplicity and low cost [53].

## Conclusion

Avian malaria parasites have a worldwide distribution, with *Plasmodium* being concentrated in South America. Hence, it is important to know the different effects of infections of these parasites on bird health and biodiversity, especially in hummingbirds, which are extremely diverse in Ecuador. The statistical analysis showed that there is not enough evidence to claim that the elevation parameter is directly related to avian malaria incidence in the current study. Either way, the outcome is consistent with the results of other studies performed in Ecuador and in Latin America. In addition, this study suggests that sex does not affect parasitemia as expected from the literature despite uniparental care of the chicks. Of the 12 bird species analyzed in this study, *H. imperatrix* is the species with the most elevated potential risk of extirpation due to its high degree of parasitemia. Therefore, understanding how avian malaria affects hummingbirds and what species are endangered by the disease is essential for the protection and conservation of Trochilidae.

## Authors’ Contributions

LMS: Conceptualization, formal analysis of the results, and writing the manuscript. LMS, MPT, and CSA: Collected samples. MPT: Contributed to the conceptualizations, identified the bird species, and revised the manuscript. CSA: Experimental assays, analysis of the results, and writing the manuscript. IRA and CSA: Performed statistical analyses. All the authors read and approved the final manuscript.
